# Evidence that CFTR is expressed in rat tracheal smooth muscle cells and contributes to bronchodilation

**DOI:** 10.1186/1465-9921-7-113

**Published:** 2006-08-28

**Authors:** Clarisse Vandebrouck, Patricia Melin, Caroline Norez, Renaud Robert, Christelle Guibert, Yvette Mettey, Frédéric Becq

**Affiliations:** 1 Institut de Physiologie et Biologie Cellulaires CNRS UMR 6187, Université de Poitiers, 40 Avenue du Recteur Pineau 86022 Poitiers, Cedex, France; 2 Laboratoire de Physiologie Cellulaire Respiratoire INSERM 0356 Université Victor Segalen Bordeaux2, 146, rue Léo Saignat, 33076 Bordeaux, Cedex, France

## Abstract

**Background:**

The airway functions are profoundly affected in many diseases including asthma, chronic obstructive pulmonary disease (COPD) and cystic fibrosis (CF). CF the most common lethal autosomal recessive genetic disease is caused by mutations of the *CFTR *gene, which normally encodes a multifunctional and integral membrane protein, the CF transmembrane conductance regulator (CFTR) expressed in airway epithelial cells.

**Methods:**

To demonstrate that CFTR is also expressed in tracheal smooth muscle cells (TSMC), we used iodide efflux assay to analyse the chloride transports in organ culture of rat TSMC, immunofluorescence study to localize CFTR proteins and isometric contraction measurement on isolated tracheal rings to observe the implication of CFTR in the bronchodilation.

**Results:**

We characterized three different pathways stimulated by the cAMP agonist forskolin and the isoflavone agent genistein, by the calcium ionophore A23187 and by hypo-osmotic challenge. The pharmacology of the cAMP-dependent iodide efflux was investigated in detail. We demonstrated in rat TSMC that it is remarkably similar to that of the epithelial CFTR, both for activation (using three benzo [*c*]quinolizinium derivatives) and for inhibition (glibenclamide, DPC and CFTR_inh_-172). Using rat tracheal rings, we observed that the activation of CFTR by benzoquinolizinium derivatives in TSMC leads to CFTR_inh_-172-sensitive bronchodilation after constriction with carbachol. An immunolocalisation study confirmed expression of CFTR in tracheal myocytes.

**Conclusion:**

Altogether, these observations revealed that CFTR in the airways of rat is expressed not only in the epithelial cells but also in tracheal smooth muscle cells leading to the hypothesis that this ionic channel could contribute to bronchodilation.

## Background

The balance between constrictor and relaxant stimuli influences the contractile state of the airway smooth muscle cell (SMC). In order to be able to propose novel therapeutic agents for the treatment of airway obstruction associated with several diseases such as asthma, chronic obstructive pulmonary disease (COPD) and cystic fibrosis (CF), it is important to understand the mechanisms underlying control of the bronchoconstriction and dilatation. Ion channels in epithelial, glandular and smooth muscle cells are of fundamental importance in regulating airway functions. Voltage-dependent cation (calcium and potassium) channels have been studied in airway SMC playing a role in excitation-contraction coupling [1]. Recently, the transient receptor potential (TRP) 6 channel has been identified in rabbit portal vein smooth muscle [2]. Different types of K^+ ^and non-selective cation channels are also present in airway SMC but their molecular entity as well as their physiological role on airway excitability and function have not been clearly established [3,4]. On the contrary, relatively little work has been carried out on the Cl^-^channels present in airway SMC. However, depolarizing Calcium/Calmodulin Dependent Protein Kinase II (CaMKII)-phosphorylated calcium-activated Cl^-^currents coupled to intracellular calcium release have been identified in tracheal myocytes [5].

Cystic fibrosis, the most common lethal autosomal recessive genetic disease, is caused by mutations of the CF gene, which normally encodes the CF transmembrane conductance regulator (CFTR), a multifunctional cAMP-dependent Cl^- ^channel in the apical membrane of secretory epithelial cells [6]. In CF, defective function of CFTR in airway epithelial cells and submucosal glands results in chronic involvement of the respiratory tract, manifested by progressive airway obstruction that begins early in life. Failure of Cl^- ^secretion through CFTR or associated ion channels results in the deshydration of endobronchial secretions. Dessicated secretions block the airways and prevent elimination of bacteria [7]. Bronchial hyper-reactivity is a common problem in CF, occurring in as many as 40% of affected individuals, which further contributes to the airway obstruction [8]. A recent study suggests possible role for Cl^- ^pathways in the modulation of airway smooth muscle function and implications for fundamental studies of airway function as well as therapeutic approaches to pulmonary disease [9].

Whereas CFTR has been generally regarded as specifically expressed in epithelial cells [6], evidence for its expression and/or function as a Cl^- ^conductance has been obtained in cardiac muscle cells [10,11], brain [12], endothelia [13,14] and more recently in aortic SMC [15,16]. In this study, we now present evidence that CFTR is also expressed in tracheal smooth muscle cells (TSMC). Exploiting new pharmacological tools (CFTR activators and inhibitors) we also provided evidence for its contribution to the bronchodilation.

## Methods

### Tissue preparation

Wistar male rats (250–300 g) were stunned and then killed by cervical dislocation according to the animal care and use local committee. Trachea were removed and placed into Krebs solution containing (in mM): 120 NaCl, 4.7 KCl, 2.5 CaCl_2_, 1.2 MgCl_2_, 1.2 KH_2_PO_4_, 15 NaHCO_3_, 11.1 D-glucose, pH 7.4.

### Cell culture

The entire trachea preparation was rapidly removed and rinced in culture medium (DMEM-HEPES supplemented with 1 % penicillin-streptomycin, 1 % Na pyruvate, 1 % non essential amino acids). Smooth muscle part of the trachea was dissected under sterile conditions in culture medium and was cut in several pieces (1–2 mm^2^). Smooth muscle pieces were placed at the bottom of individual wells of 6-well culture plates containing culture medium enriched with 10 % foetal calf serum (FCS). Organ culture plates were placed in a humidified incubator at 37°C under 5% CO_2 _in air. The medium was changed every 48 h. After one week, confluent cells were rinsed twice with Hanks' balanced salt solution and then passaged with trypsin-EDTA. Isolated cells were then seeded in a 24-well culture plate for functional study of chloride channels activity. Cells were left in culture medium for 48 h before they were growth arrested using serum-free culture medium supplemented with 1% insulin-transferrin-selenium (ITS) (as previously described) [17].

### Immunofluorescence study

Cells grown on glass coverslips were washed 3 times in Tris-buffered saline (TBS) and after fixation, non specific binding sites were blocked with TBS containing 0.5% BSA and 0.05% saponin for 1 h. Cells were incubated with an anti-CFTR C-terminal monoclonal antibody (1:100, Ig2a, mouse anti-human, R&D Systems, Minneapolis, MN, USA) or with monoclonal anti-alpha smooth muscle actin (1:200, IgG2a Cy3 conjugate, Sigma Chemicals, St Louis, MO, USA) for 2 h at room temperature. After 3 washes, cells were incubated with the FluoProbes 488 (1:400, Interchim, Montluçon, France) secondary antibody. In the control, the primary antibody was omitted. Nuclei were stained in blue with TO-PRO-3 iodide (Molecular Probes, Eugene, OR) for 15 min at room temperature (1:1000 in TBS). Fluorescence was examined with a spectral confocal station FV 1000 installed on an inverted microscope IX-81 (Olympus, Tokyo, Japan).

### Iodide efflux

CFTR Cl^- ^channel activity was assayed by measuring the rate of iodide (^125^I) efflux from cells as previously described [18]. All experiments were performed with a MultiPROBE^®^IIex robotic liquid handling system (Perkin Elmer Life Sciences, Courtaboeuf, France). At the beginning of each experiment, cells were washed twice with efflux buffer containing (in mM) 136.9 NaCl, 5.4 KCl, 0.3 KH_2_PO_4_, 0.3 NaH_2_PO_4_, 1.3 CaCl_2_, 0.5 MgCl_2_, 0.4 MgSO_4_, 5.6 glucose and 10 HEPES, pH 7.4. Cells were incubated in efflux buffer containing Na^125^I (1 μCi Na^125^I/ml, NEN, Boston, MA) during 1 h at 37°C. Cells were then washed with efflux medium to remove extracellular^125^I. The loss of intracellular^125^I was determined by removing the medium with efflux buffer every 1 min for up to 10 min. The first three aliquots were used to establish a stable baseline in efflux buffer alone. A medium containing the appropriate drug was used for the remaining aliquots. Residual radioactivity was extracted with 0.1 N NaOH/0.1% SDS, and determined using a Packard Cobra™II gamma counter (Perkin Elmer life Sciences, Courtaboeuf, France). The fraction of initial intracellular^125^I lost during each time point was collected and time-dependent rates of^125^I efflux calculated from: ln (^125^I_t1 _/^125^I_t2_)/(t_1 _– t_2_) where^125^I_t _is the intracellular^125^I at time t, and t_1 _and t_2 _successive time points [18]. Curves were constructed by plotting rate of^125^I versus time. All comparisons were based on maximal values for the time-dependent rates (k = peak rates, min^-1^) excluding the points used to establish the baseline (k peak-k basal, min^-1^) [18]. All inhibitors were pre-incubated 30 min.

### Contraction measurement on isolated tracheal rings

After separation of connective tissues, the trachea was cut into rings of 3 mm length. Tracheas were mounted between a fixed clamp at the base of a water-jacketed 5 ml organ bath containing an oxygenated (95% O_2 _and 5% CO_2_) Krebs solution and an IT1-25 isometric force transducer (Emka Technologies, Paris, France) [15,16]. All experiments were performed at 37°C. A basal tension of 2 g was applied in all experiments. During 1 h, tissues were rinsed three times in Krebs solution and the basal tone was always monitored and adjusted to 2 g. 1 μM Carbachol (CCH) were used to evoke the sustained contractile response. Once the sustained tension was established, the tissues were allowed to equilibrate further for 30 min before cumulative addition of agonist to the bath. Cumulative concentration-response relationships for the relaxant effect of MPB compounds were determined in trachea rings following stable contraction. The relaxant effect of CFTR agonists was expressed as percentage contraction of the agonist-constricted tracheal rings. IC_50 _was calculated as the drug concentration inducing a half-maximal dilatation (or inhibition of contraction).

### Statistics

Results are expressed as means ± SEM of n observations. Sets of data were compared with analysis of variance (ANOVA) or a Student's *t *test. Differences were considered statistically significant when *P *< 0.05. ns: non significant difference, * P < 0.05, ** P < 0.01, *** P < 0.001. All statistical tests were performed using GraphPad Prism version 4.0 for Windows (Graphpad Software, San Diego, CA) and Origin version 5.0 (Rockware, Golden, CO).

### Drugs and chemical reagents

The benzo [*c*]quinolizinium compounds 10-chloro-6-hydroxybenzo [*c*]quinolizinium chloride (MPB-07), 5-butyl-10-chloro-6-hydroxybenzo [*c*]quinolizinium chloride (MPB-91) and 10-fluoro-6-hydroxybenzo [*c*]quinolizinium chloride (MPB-80) were prepared as described previously [19]. Carbamylcholine, glibenclamide, Insulin – Transferrin – Selenium (ITS), monoclonal antibody anti-α-smooth muscle actin, Na pyruvate, non essential amino acids were purchased from Sigma Chemicals (Saint Quentin Fallavier, France). DMEM-HEPES, Foetal Calf Serum (FCS), Penicillin-Streptomycin, trypsin-EDTA and Hanks' balanced salt solution were purchased from Gibco (Invitrogen Corporation, Cergy Pontoise, France). CFTR_inh_-172 was purchased from Calbiochem (USA). All drugs were prepared in dimethyl sulfoxide (DMSO) except MPB-07, MPB-80 and carbachol that were prepared as stock solution in distilled water. The maximal concentration of DMSO used in experiments was 0.1 % for glibenclamide and 0.3 % for MPB-91 and had no effect on mechanical activity of the rings.

## Results

### Analysis of chloride transports in organ culture of rat TSMC

**Figure 1:**
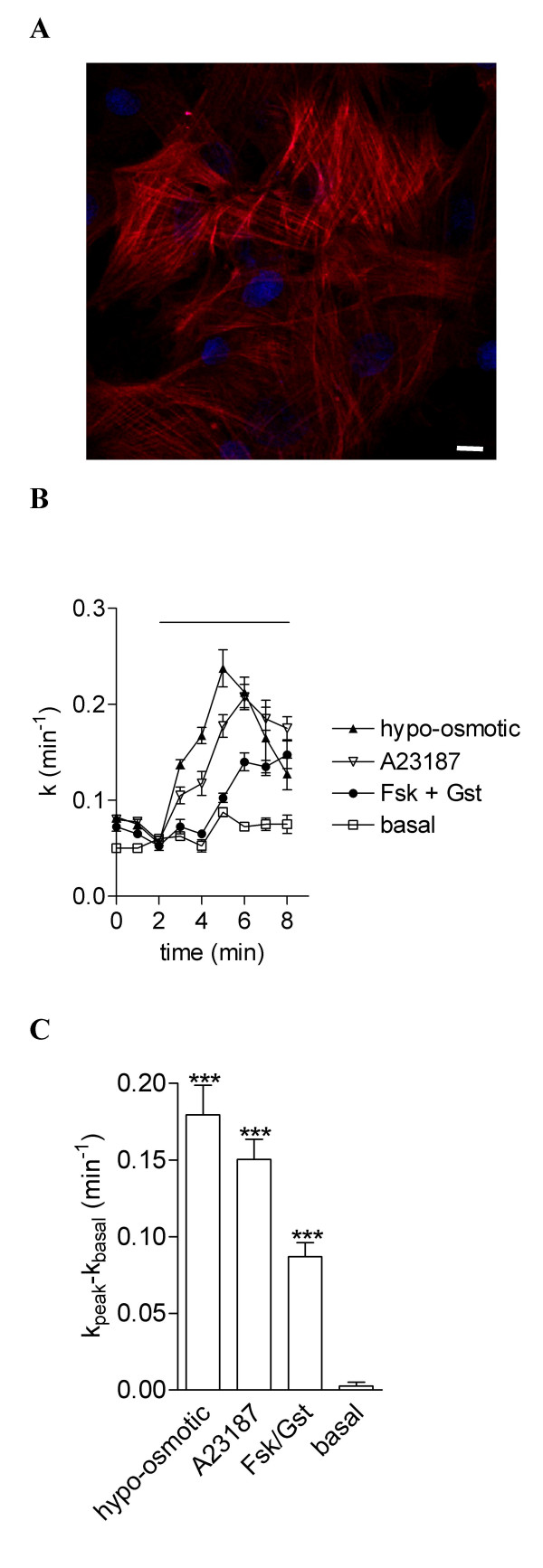
**Analysis of chloride transports in rat airway smooth muscle cells**. **A **Immunofluorescence study of α-smooth muscle actin in tracheal smooth muscle cells from rat and **B **in the absence of primary antibody. Scale bars are 10 μm. **C **The stimulation of iodide efflux as a function of time was evoked by an hypo-osmotic challenge, 1 μM A23187 and cAMP agonists (10 μM forskolin + 30 μM genistein) in rat airway smooth muscle cells as compared to basal. **D **Summary of the relative rates presented as mean ± S.E.M. Basal was vehicle alone.

Immunostaining with the monoclonal anti-α-smooth muscle actin antibody was positive (Fig. 1A) for all cells demonstrating the presence of an homogenous population of smooth muscle cells. The cells were positive for the contractile phenotype. No staining was detected when the anti-α-smooth muscle actin antibody was omitted (data not shown). To investigate the Cl^- ^transports is tracheal smooth muscle cells, we prepared organ culture of rat tracheal smooth muscle cells (TSMC). We began our study by examining three different pathways for Cl^- ^channels stimulation, i.e. cAMP-, Ca^2+^- and volume-dependent pathways. Transport properties of rat TSMC were studied using the iodide efflux method allowing rapid and efficient Cl^- ^transport investigation [15,16], stimulated in response to cell exposure to the cAMP-agonist forskolin with the isoflavone genistein, to the Ca^2+ ^ionophore A23187 and to hypo-osmotic bath solution. No significant iodide efflux was measured in resting TSMC (Fig. 1B, open squares noted basal). However, a significant stimulation (*P *< 0.001) of iodide efflux by the hypo-osmotic challenge (n = 4), A23187 (1 μM, n = 4) and cAMP agents (10 μM forskolin with 30 μM genistein, n = 4) was obtained as compared to resting TSMC cells (n = 4) (Fig. 1C). The response of cells to hypo-osmotic solution and A23187 was faster and more pronounced than that of forskolin/genistein (Fig. 1C). This first set of results shows multiple Cl^- ^transports stimulated by hypo-osmotic challenge, cAMP and Ca^2+ ^agonists in rat TSMC.

### Does CFTR underlie the cAMP-dependent chloride transport in rat TSMC?

**Figure 2:**
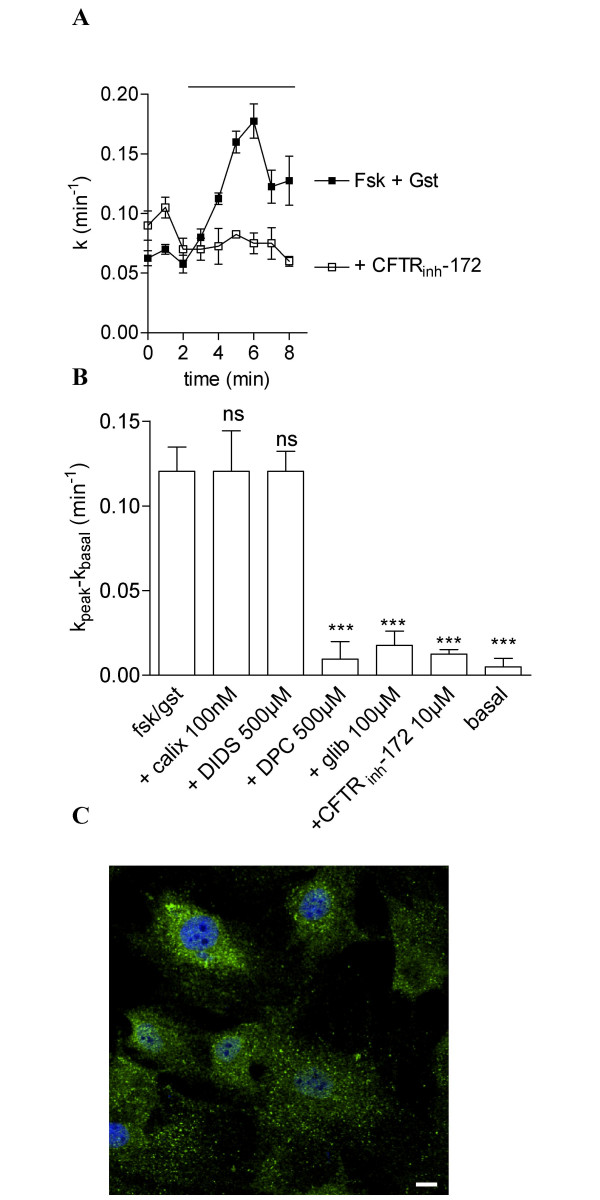
**Pharmacological inhibition and immunolocalisation of CFTR in rat tracheal smooth muscle cells**. **A **Iodide efflux response as a function of time evoked by cAMP agonists (10 μM forskolin + 30 μM genistein) and 10 μM CFTR_inh_-172. **B **Effect of 100 μM glibenclamide, 500 μM DPC, 500 μM DIDS, 100 nM calixarene and 10 μM CFTR_inh_-172 on the efflux stimulated by cAMP agonists in rat tracheal smooth muscle cells as indicated. Basal was vehicle alone. Data are presented as mean ± S.E.M. All experimental conditions have been repeated: n = 4. **C **Immunofluorescence study of CFTR in TSMC and **D **in the absence of primary antibody. α-smooth muscle actin is stained in red, CFTR in green and nucleus (TOPRO-3 staining) in blue. Scale bars are 10 μm.

We then decided to focus our study on the cAMP-dependent Cl^- ^transport because it has never been described in tracheal smooth muscle. It is known from numerous studies that in epithelial cells, the main Cl^- ^channel underlying cAMP-dependent Cl^- ^transport is the CFTR Cl^- ^channel [6-9]. In the next series of experiments we hypothesized that CFTR is present and functional in TSMC by using the cocktail forskolin/genistein (Fig. 2A). To test this hypothesis, we first used different classes of Cl^- ^channels inhibitors to characterize the cAMP-dependent Cl^- ^transport in this preparation. Glibenclamide and diphenylamine-2-carboxylic acid (DPC) are two non-specific inhibitors of Cl^- ^channels including CFTR [16,20,21], the stilbene derivative DIDS is a general blocker of Cl^- ^channels but does not inhibit CFTR from the extracellular [16,20,21] and TS-TM calix [4]arene is an inhibitor of outwardly rectifying Cl^- ^channels but not of CFTR [16,21,22]. We observed that 100 μM glibenclamide or 500 μM DPC fully inhibited the stimulation of iodide efflux with forskolin/genistein while neither 100 nM calixarene nor 500 μM DIDS have an inhibitory effect (Fig. 2B, n = 4 for each). To confirm this pharmacological profile of inhibition, we tested the thiazolidinone compound CFTR_inh_-172 which has been recently developed as a specific CFTR blocker with no significant inhibitory action on other Cl^- ^channels, and especially on the volume- and calcium-activated Cl^- ^channels [23]. We compared the iodide efflux response of rat TSMC to the cocktail forskolin/genistein in the presence or absence of CFTR_inh_-172 used at 10 μM. It is clear from the results presented in Fig. 2A and 2B, that the compound fully inhibited the efflux demonstrating that CFTR is likely to be responsible for most of the cAMP-regulated Cl^- ^transport in TSMC. This profile of inhibition is thus in perfect agreement with that determined for CFTR in epithelial [20,21] and aortic vascular smooth muscle cells [15,16] and further supports our hypothesis that CFTR is present, functional and cAMP-regulated in tracheal myocytes. To study the presence of CFTR in rat TSMC, CFTR localization was performed by indirect immunofluorescence confocal microscopy. By using anti-CFTR C-terminal monoclonal antibody, CFTR protein was detected in the plasma membrane and within cytoplasmic compartments of rat TSMC (Fig. 2C). No staining was detected when the primary antibody was omitted (data not shown).

### Pharmacological activation of *CFTR *channels in rat TSMC

**Figure 3:**
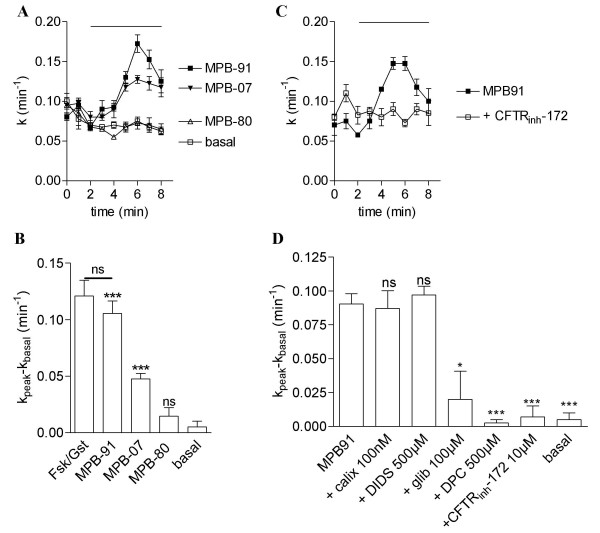
**Pharmacological activation of CFTR chloride channels activity in rat airway smooth muscle cells**. **A **Iodide efflux responses as a function of time evoked by 250 μM MPB-07, MPB-91 and MPB-80. All experimental conditions have been repeated: n = 4. Basal was vehicle alone. **B **Summary of the data for each experimental condition after stimulation by MPB-07, MPB-80, MPB-91. **C **Iodide efflux response as a function of time evoked by MPB-91 (250 μM) and 10 μM CFTR_inh_-172. **D **Effect of 100 μM glibenclamide, 500 μM DPC, 500 μM DIDS, 100 nM calixarene and 10 μM CFTR_inh_-172 on the efflux stimulated by MPB-91 (250 μM) in rat tracheal smooth muscle cells as indicated. Basal was vehicle alone. Data are presented as mean ± S.E.M. All experimental conditions have been repeated: n = 4. *** P < 0.001, * P < 0.05. ns: non-significant difference.

If CFTR in TSMC and in epithelial cells are similar, then we should observe its stimulation using known pharmacological activators of the epithelial CFTR, such as the benzo [*c*]quinolizinium derivatives MPB-07 and MPB-91 [15,16,19,24]. A third drug within this chemical family, MPB-80, not able to stimulate CFTR channel activity [15,19] was also used here. MPB-07 and MPB-91 (250 μM, n = 4, Fig. 3A, B) stimulated iodide efflux from rat TSMC but no stimulation was observed with MPB-80 (250 μM, n = 4, Fig. 3A, B). Like with the agonists forskolin/genistein, the iodide efflux stimulated by MPB-91 was insensitive to 100 nM calixarene, 500 μM DIDS but fully inhibited in the presence of 100 μM glibenclamide, 500 μM DPC and 10 μM CFTR_inh_-172 (Fig. 3C, D). Therefore, given the pharmacological profile of activation using either forskolin/genistein or the benzo [*c*]quinolizinium CFTR activators (MPB-91>MPB-07) and the fact that the inactive compound MPB-80, like for the epithelial and aortic CFTR [15,19], is not able to stimulate iodide efflux in TSMC, these results demonstrate that CFTR in TSMC shares numerous pharmacological properties with that determined for epithelial and aortic CFTR [15,16,20,21].

### Role of CFTR in agonist-dependent bronchodilation of smooth muscle cells

**Figure 4:**
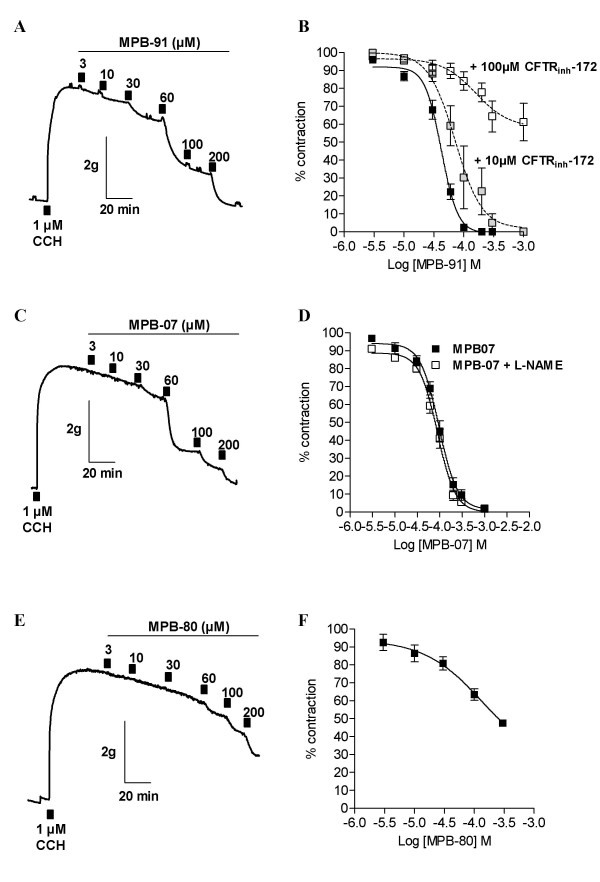
**Bronchodilation effect of CFTR activators on rat trachea**. Typical traces from experiments performed with tracheal rings preconstricted with 1 μM CCH. **A **The effect on tension of various concentrations of MPB-91. **B **Concentration-dependent curves are displayed, showing the bronchodilation of tracheal rings preconstricted by 1 μM CCH for MPB-91 in absence (IC_50 _= 42 ± 5 μM, n = 13) or in presence of 10 μM CFTR_inh_-172 (IC_50 _= 71 ± 3 μM, n = 6), or 100 μM CFTR_inh_-172 (IC_50 _> 150 μM, n = 7). **C **The effect on tension of various concentration of MPB-07. **D **Concentration-dependent curves showing the bronchodilation of tracheal rings preconstricted by 1 μM CCh for MPB-07 in absence (IC_50 _= 94 ± 4 μM, n = 16) or presence (IC_50 _= 87 ± 5 μM, n = 15) of 100 μM L-NAME (pre-incubated 30 min). **E **The effect on tension of various concentrations of MPB-80. **F **Concentration-dependent curves are displayed, showing the bronchodilation of tracheal rings preconstricted by 1 μM CCH for MPB-80 (IC_50 _= 135 ± 5 μM, n = 8).

In the last part of our study, we performed experiments on rat tracheal rings mounted in an organ bath apparatus and measured their muscular activity. Initial experiments were carried out to evaluate the contractile response to carbachol (CCH). We obtained a concentration-response curve for CCH used between 10^-7 ^to 10^-3 ^M and determined a half maximal response EC_50 _of 10^-6 ^M (n = 8). This concentration of CCH was then used in the next experiments described below. The CCH-induced constriction reached a maximum, indicated by a plateau phase, and then declined slowly during 4 h. The vehicle DMSO (used at maximum 0.1%) had no effect on the maximum response (data not shown). We applied the benzoquinolizinium CFTR activators MPB-91, MPB-07 and the inactive analogue MPB-80 via cumulative application into the organ bath (3–200 μM). Clearly, MPB-91 induced a concentration-dependent relaxation of rat tracheal ring preconstricted by 1 μM CCH (Fig. 4A) that began at 10 μM and was complete for 200 μM. From 13 different tracheal rings we determined half-maximal relaxation value IC_50 _for the CFTR activators MPB-91; IC_50 _of 42 ± 5 μM (n = 13) (Fig. 4B). Very similar results were obtained with the other CFTR activator, MPB-07 (Fig. 4C) (IC_50 _of 94 ± 4 μM, n = 16) (Fig. 4D). Pre-incubation of N^G^-nitro-L-arginine methyl ester (L-NAME), an inhibitor of nitric oxide synthase (NOS), occurred before MPB07 exposure in constricted airway segments from rat (Fig 4D) has no significant effect on relaxation. This result suggests an epithelium-independent relaxation of rat TSMC. A very different effect was obtained with MPB-80 (the inactive analogue of MPB-91). Indeed with MPB-80 a small relaxant effect was only seen above 60 μM (Fig. 4E). Using 8 different tracheal rings we failed to relax more than 50% of the initial constriction induced by 1 μM CCH. Because complete relaxation was not obtained we did not calculate IC_50 _value (Fig. 4F, n = 8 for each concentration). To establish the implication in MPB-dependent bronchodilation, we evaluated the effect of the specific CFTR inhibitor CFTR_inh_-172 as antagonist of MPB-91. Fig. 4B shows that in the presence of 10 μM and 100 μM CFTR_inh_-172 the concentration response of MPB-91 shifted to the right indicating concentration-dependent inhibition of CFTR_inh_-172. Surprisingly, we observed that the response induced by MPB-80 (Fig 4E) was similar to the response observed after inhibition of CFTR activity using 100 μM CFTR_inh_-172 in presence of MPB-91 (Fig 4B), a concentration which fully inhibited the CFTR activity (see effect of 10 μM CFTR_inh_-172 on efflux Fig 2B and 3D). These results suggest that the relaxation induced by MPB-80 correspond to a CFTR-independent effect on rat TSMC.

## Discussion

The present study demonstrates for the first time that rat tracheal smooth cells express CFTR chloride channel and that its activation leads to a CFTR_inh_-172 dependent bronchodilation after muscarinic contraction. Based on our experiments, a number of important findings could be summarized as follow:

(i) three different Cl^- ^transports stimulated by cAMP, intracellular calcium and cell volume are present in rat TSMC in organ culture,

(ii) the activation by the pharmacological CFTR activators MPB derivatives of the tracheal CFTR channel is remarkably similar to that of the epithelial CFTR. Moreover, the structural and pharmacological specificity of benzo [*c*]quinolizinium agents (i.e. the different activity of MPB-80, MPB-07 and MPB-91) are conserved for CFTR in epithelia and TSMC,

(iii) the inhibitory profile (glibenclamide, DPC and CFTR_inh_-172) of the tracheal CFTR channel activity is identical to that of the epithelial CFTR suggesting that CFTR is the major pathway for cAMP-regulated chloride transport in TSMC,

(iv) finally, using isometric contraction measurement on rat isolated tracheal rings, we found that the activation of CFTR leads to bronchodilation with a concentration-dependent inhibition by CFTR_inh_-172.

The airway is a complex system with more than 20 different cell types, a smooth muscle layer surrounding an epithelial layer facing the lumen. In this multicellular organ, CFTR is functionally expressed both in epithelial [7] and in smooth muscle cells (this study). However, the link between CFTR-mediated ion transport and the lung physiology has been the subject of intense debate and remains poorly understood. The role of the airway epithelium in modifying the contractility of the underlying smooth muscle has been suggested but is not yet fully demonstrated. For example, it has been suggested that the primary function of the epithelium is to provide a barrier of protection between the airway smooth muscle and inhaled irritants [25]. Other studies have demonstrated that the epithelium can be an active source of mediators that relax constricted airways [26-28]. Fortner et al. [9] tested the hypothesis that the epithelium-dependent relaxation to agonists, like substance P and ATP, depends on the activity of chloride channels. They have shown a possible role for chloride pathways in the modulation of airway smooth muscle function using various chloride channel inhibitors. This work has also demonstrated that the relaxation to substance P and ATP persisted in the tracheas from *Cftr*^-/- ^mice, and that the magnitude of the relaxation was not significantly different from that in the wild-type animals. This indicated that CFTR function is not required for airways relaxation to substance P and ATP [9]. However, another study demonstrated that tracheas from CF mice have impaired relaxation in response to electrical field stimulation [29]. This effect appears to be related to the lack of NO produced by CF respiratory epithelium and is readily reversible with exogenous NO or L-arginine [29]. This observation is consistent with other findings showing decreased exhaled NO in patients with CF [30-32] and a reduction in NOS expression in CF murine and human airway epithelial cells [33].

Recent progress into the pharmacology of chloride channels provided interesting new tools to study the contribution of these transport proteins into organ physiology. We took advantage of the specific CFTR inhibitor CFTR_inh_-172 [23] to monitor the muscular reactivity of isolated rat tracheal rings. Using this agent we found two complementary effects. First, it fully inhibits the iodide efflux stimulated by forskolin/genistein or MPB compounds, indicating that CFTR is the major ionic channel responsible for the cAMP-regulated Cl^- ^transport in TSMC. Second, we found concentration-dependent inhibition by CFTR_inh_-172 of the bronchodilation induced by the CFTR activators MPB after muscarinic stimulation. This pharmacological evidence is in favour of an unexpected role of CFTR in bronchodilation. It is well known that airway smooth muscle relaxation is brought about predominantly by stimulation of adenyl cyclase-coupled receptors (e.g. β2-adrenoceptor) resulting in elevation of cell cyclic adenosine monophosphate content. Importantly, this signalling pathway is central in activating CFTR-mediated chloride transport in epithelial [7,21], aortic [15], and airway smooth muscle cells. Taken together these results illuminate a direct implication for CFTR in the bronchodilation of the rat trachea.

In disorders of the conducting airways like asthma, COPD and CF, understanding the molecular mechanisms controlling the contractile state of the airway smooth muscle cell may generate new therapeutic opportunities. In CF, chronic endobronchial infection is a primary feature of the pulmonary disease. In addition, defective function of CFTR in airway epithelial cells and submucosal glands results in chronic involvement of the respiratory tract, manifested by progressive airway obstruction that begins early in life [7,36]. Asthma pathogenesis is characterized by progressive airway wall remodelling that includes, in part, local inflammation and fibrosis as well as increased airway smooth muscle mass [34]. Recently, Hays et al. [35] demonstrated structural changes to airway smooth muscle in CF. They shown increased smooth muscle content of the airway in subjects with CF compared to healthy controls. This increase is due to smooth muscle cell hyperplasia without hypertrophy [35]. These findings imply that smooth muscle cell proliferation is a characteristic of airway remodelling in CF [35]. Also, in the lungs of *Cftr *null mice, an alteration in airway neuroendocrine cell and neural components has been proposed [37]. It is characterized by a decreased density of airway smooth muscle innervation, mass and neuromuscular junctions [37]. These observations could suggest that CFTR plays a role in the development of pulmonary neuroendocrine cell system, lung innervation and airway smooth muscle. Although further studies will be required, these numerous informations, together with our finding of CFTR expression in airway smooth muscle, suggest that CFTR in the airways may have complex functions depending on the cell type in which it is functional as a chloride channel. Nevertheless, with greater understanding of the molecular mechanisms leading to the control of bronchodilation and the identification of novel bronchodilators(e.g. potent CFTR activators) we are likely to be able to develop new therapeutics for individuals with airway disease.
